# Mode of Death on Chagas Heart Disease: Comparison with Other Etiologies. A Subanalysis of the REMADHE Prospective Trial

**DOI:** 10.1371/journal.pntd.0002176

**Published:** 2013-04-25

**Authors:** Silvia M. Ayub-Ferreira, Sandrigo Mangini, Victor S. Issa, Fátima D. Cruz, Fernando Bacal, Guilherme V. Guimarães, Paulo R. Chizzola, Germano E. Conceição-Souza, Fabiana G. Marcondes-Braga, Edimar A. Bocchi

**Affiliations:** Heart Institute (InCor) do Hospital das Clínicas da Faculdade de Medicina da Universidade de São Paulo, São Paulo, Brazil; Hospital Universitário, Brazil

## Abstract

**Background:**

Sudden death has been considered the main cause of death in patients with Chagas heart disease. Nevertheless, this information comes from a period before the introduction of drugs that changed the natural history of heart failure. We sought to study the mode of death of patients with heart failure caused by Chagas heart disease, comparing with non-Chagas cardiomyopathy.

**Methods and results:**

We examined the REMADHE trial and grouped patients according to etiology (Chagas *vs* non-Chagas) and mode of death. The primary end-point was all-cause, heart failure and sudden death mortality; 342 patients were analyzed and 185 (54.1%) died. Death occurred in 56.4% Chagas patients and 53.7% non-Chagas patients. The cumulative incidence of all-cause mortality and heart failure mortality was significantly higher in Chagas patients compared to non-Chagas. There was no difference in the cumulative incidence of sudden death mortality between the two groups. In the Cox regression model, Chagas etiology (HR 2.76; CI 1.34–5.69; p = 0.006), LVEDD (left ventricular end diastolic diameter) (HR 1.07; CI 1.04–1.10; p<0.001), creatinine clearance (HR 0.98; CI 0.97–0.99; p = 0.006) and use of amiodarone (HR 3.05; CI 1.47–6.34; p = 0.003) were independently associated with heart failure mortality. LVEDD (HR 1.04; CI 1.01–1.07; p = 0.005) and use of beta-blocker (HR 0.52; CI 0.34–0.94; p = 0.014) were independently associated with sudden death mortality.

**Conclusions:**

In severe Chagas heart disease, progressive heart failure is the most important mode of death. These data challenge the current understanding of Chagas heart disease and may have implications in the selection of treatment choices, considering the mode of death.

**Trial Registration:**

ClinicalTrails.gov NCT00505050 (REMADHE)

## Author Summary

Chagas disease remains a burden for public health systems in Latin American countries. Several authors believe that sudden death is the main cause of death in this population. So many efforts have been made to prevent sudden death in Chagas disease. In order to verify if sudden death is the leading cause of death in Chagasic heart failure, we performed a subanalysis of the REMADHE prospective trial, which included a population of outpatients in a tertiary referral center for heart failure. We grouped patients according to etiology (Chagas *vs* non-Chagas) and modes of death that were classified as progressive heart failure death, sudden death, other cardiovascular death, noncardiovascular death or unknown death. Our study showed that in this end of the spectrum of presentation of Chagas disease, with systolic heart dysfunction, progressive heart failure is the main mode of death. These data have implications for the development of new strategies for prevention of chagasic heart failure.

## Introduction

Chagas disease (*American trypanosomiasis*) remains a burden for public health systems in Latin American countries [1]. In fact, the global prevalence of Chagas disease has reached 9 million people and 25 million are estimated to be at risk worldwide [2]. As a result of globalization and migration, non-endemic countries have reported an increase in the prevalence of Chagas disease. It has been estimated that 47,000 *T. cruzi*-infected persons are now living in Spain and another 300,000 in the United States [3].

Sudden death has been considered the main cause of death in patients with Chagas heart disease [4]. Nevertheless, this information comes mainly from a period before the introduction of drugs that changed the natural history of heart failure such as beta-blockers, angiotensin-converting enzyme inhibitors (ACEI) and angiotensin II receptor blockers (ARB). Furthermore, many studies that support this finding include heterogeneous cohorts of patients, in particular patients without ventricular dysfunction [4].

To the best of our knowledge there is not a recent publication studying the mode of death in Chagas heart disease. The management of Chagas heart disease is complex and includes the treatment of complex arrhythmias and heart failure. The precise understanding of current natural history of patients presenting systolic dysfunction is indispensable to plan adequate health politics and improve survival. Therefore, we sought to study the mode of death of patients with heart failure caused by Chagas heart disease, comparing with non-Chagas cardiomyopathy.

## Materials and Methods

### Patients

We examined the patients included in the REMADHE (**R**epetitive **E**ducation at Six-Month Intervals and **M**onitoring for **A**dherence in **He**art Failure Outpatients) trial; For the purpose of current study, patients were grouped according to etiology (Chagas vs non-Chagas groups) and mode of death.

**Figure 1:**
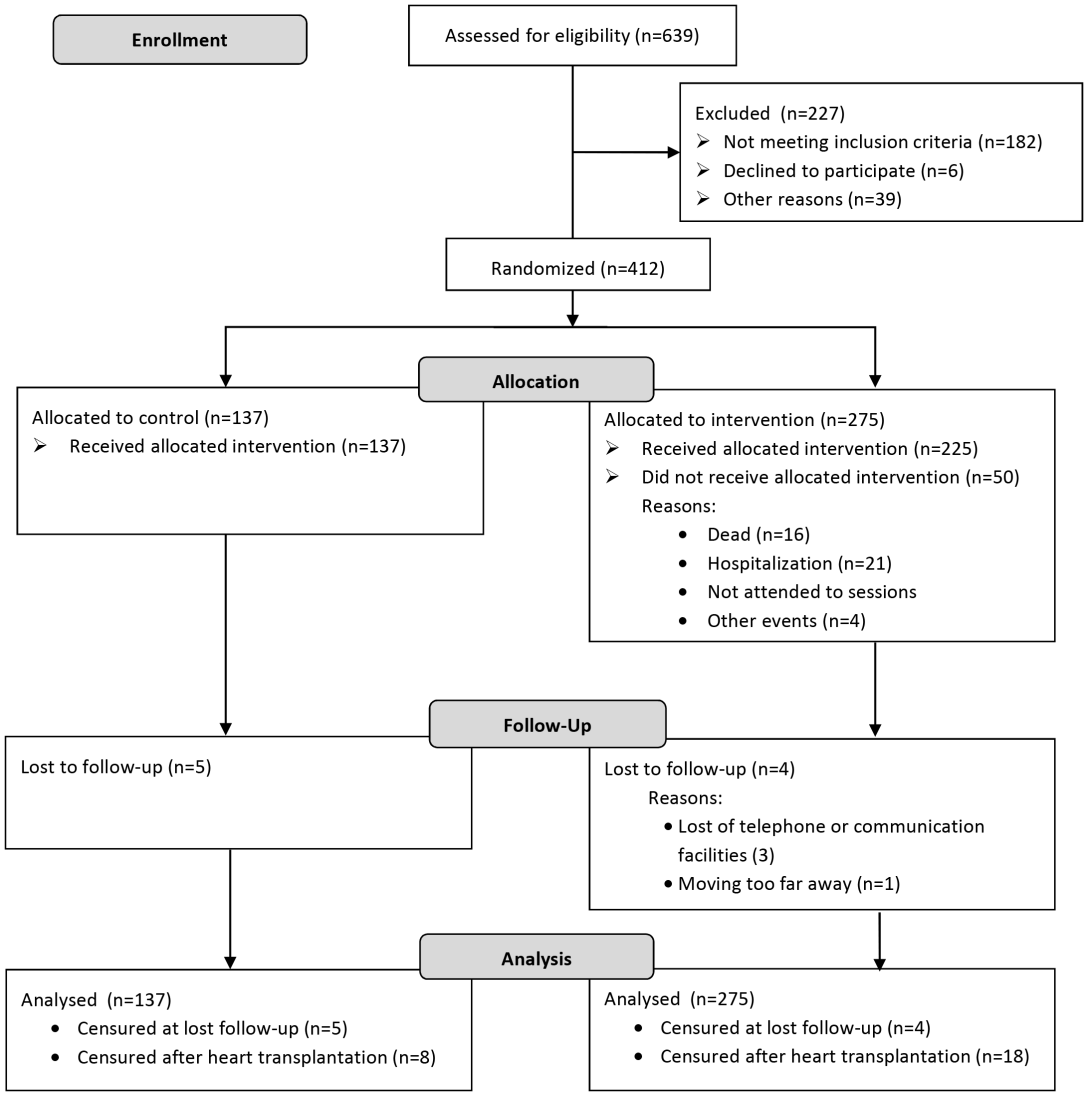
Flow diagram of the REMADHE trial.

REMADHE is a prospective, randomized, single-center open parallel trial controlled by nonintervention simple randomization and designed to compare a disease management program versus control in patients with chronic heart failure. Patients enrolled in the study were under ambulatory care in a tertiary referral center and were followed by a cardiologist with experience in heart failure. Inclusion elapsed from October 1999 until January 2005. Patients were aged 18 years or older with irreversible chronic heart failure of at least 6-months. Exclusion criteria included patients' inability to attend educational sessions and researchers' inability to monitor patients because of the patients' lack of transportation, social or communication barriers; myocardial infarction or unstable angina within 6 months before randomization; cardiac surgery or angioplasty within 6 months before randomization; hospitalized patients or recently discharged patients; any severe systemic disease that could impair expected survival; procedures that could influence follow-up; pregnancy or childbearing potential (figure 1). The REMADHE trial demonstrated that a disease management program was associated with reduction in unplanned hospitalization, total hospital days and need for emergency care, as well as improved quality of life [5], [6].

### Definitions

Patients were divided into two groups: Chagas and non-Chagas. The diagnosis of Chagas disease was based on epidemiological information along with serological tests (indirect immunofluorescence, passive haemaglutination, immunoenzymatic assay) positive for *Trypanosoma cruzi* [7]. Patients with an alternative diagnosis, or a mixed etiology for the cardiomyopathy were included in non-Chagas group.

All deaths were classified according to specified definitions. The agreement of two members of the study on the cause of death was mandatory. Sudden death was defined as an unexpected death occurred within 1 hour of the onset of new symptoms or occurred unwitnessed in a previously stable patient [8].

Heart failure death was defined as a death that occurred because of worsening or intractable heart failure, which included cardiogenic shock, pulmonary edema and terminal arrhythmias during hospital stay for aggravated heart failure.

Other cardiovascular death was defined as a death that occurred after a cerebrovascular accident, vascular disease, myocardial infarction or cardiovascular procedure. Cerebrovascular accident was defined as a persistent disturbance of neurological function. Diagnosis required characteristic history, physical examination, imaging techniques and/or autopsy data.

Myocardial infarction (MI) death was defined as a death that occurred after a verified acute MI. Noncardasiovcular death was defined as a death resulting from a noncardiac reason. Unknown death was defined as a death that did not have a definitive cause.

Patients were categorized according to the New York Heart Association functional class [9] during evaluation of the medical staff of our heart failure clinic.

### End Point

The primary end-point of the study was all-cause, heart failure and sudden death mortality that were obtained during follow-up, either from the trial database, from review of medical records or by telephone contact with family members. Additionally, Cox proportional-hazards regression models were used to explore the relationship of mode of death, etiology and other variables with survival time. For Cox proportional hazards model, each mode of death is looked at in turn and all other modes of death are censored.

### Statistical Analysis

Results are presented as frequencies, mean (SD), or median (interquartile range), as appropriate. For effects of group comparison, the *t* test was used for normal distribution and Mann-Whitney test was used to compare variables without normal distribution. For categorical variables, chi-square test or the Fisher exact test was applied. Survival was estimated by the Kaplan–Meier method, and differences in survival between groups were assessed by the log-rank test. Cox proportional-hazards models were used to compare the rates of deaths for each mode of mortality. In the analysis, data on patients was censored at the time of implantation of a defibrillator or the time of heart transplantation. All analyses and graphs were performed with SPSS statistical software version 13.0 and Graphpad Prism software version 5.0.,

### Ethics Statement

The study protocol was approved by the institutional ethics committee of Hospital das Clinicas da Faculdade de Medicina da Universidade de Sao Paulo and all patients provided written informed consent. All data analyzed were anonymized.

## Results

A total of 412 patients were enrolled in the REMADHE trial. For the purpose of the current study, we excluded patients with left ventricular ejection fraction ≥50% (60 patients) and those who had ICD (10 patients) and we censored nine patients at the time of implantation of a defibrillator. Thus, 342 patients were analyzed. The first inclusion occurred in October 1999, and patients were followed until February 2010 with a mean follow-up of 1,284 days±895 days. Despite our cohort included 342 patients, we have information about New York Heart Association functional class (NYHA FC) only in 296 patients. Baseline characteristics of the patients are described in table 1.

**Table 1:** Characteristics of the study population.

	Total	Chagas	Non-Chagas	ρ
Number of patients (%)	342	55 (16.1)	287 (83.9)	
Age, median (IQR), years	50 (44–58)	49 (42–55)	50 (44–58)	0.316
Male (%)	68.4	56.4	70.7	0.036
BMI, median (IQR), kg/m^2^	25.0 (22.3–28.4)	23.5 (21.3–26.4)	25.3 (22.5–29.0)	0.003
NYHA class*(%)				
I/II	60.8	56.5	61.6	0.517
III/IV	39.2	43.5	38.4	0.517
Disease Management Program Group (%)	67.7	65.5	66.9	0.835
LVEDD, median (IQR), mm	70 (63–77)	67 (63–71)	70 (64–77)	0.006
Etiology: Ischemic/Idiopathic/Others (%)			29/27/44	
LVEF (%)	32 (26–37)	34 (28–40)	31 (25–37)	0.015
Hemoglobin, median (IQR), g/dl	14.1 (12.9–15.1)	13.9 (12.6–14.9)	14.1 (13.0–15.2)	0.321
CrCl, median (IQR), ml/min/1,73 m^2^	71.2 (53.5–93.1)	64.0 (49.3–86.4)	72.0 (54.1–93.6)	0.128
Serum sodium, median (IQR), mEq/l	139 (137–141)	138 (136–140)	139 (137–141)	0.700
HF Pharmacotherapy (%)				
Beta-Blocker	73.0	38.2	79.8	<0.001
ACEI/ARB	95.5	96.4	95.4	0.749
Spironolactone	60.4	67.3	59.0	0.251
Warfarin	18.4	16.4	18.8	0.667
Amiodarone	9.1	12.7	8.4	0.306

Abbreviations: ACEI, angiotensin-converting enzyme inhibitor; ARB, angiotensin II receptor blocker; BMI, body mass index; CrCl, creatinine clearance; NYHA, New York Heart Association functional class; IQR, interquartile range; LVEF, left ventricular ejection fraction; LVEDD, left ventricular end diastolic diameter.

* Information available in 296 patients.

As compared to non-Chagas, Chagas patients had lower body mass index, smaller end-diastolic left ventricle diameter, and smaller proportion of patients under beta-blocker therapy. Chagas patients also had higher proportion of females and larger left ventricular ejection fraction (Table 1).

Chagas patients had a higher incidence of death related to cerebrovascular accident and non-cardiovascular deaths. They also had a higher number of hospitalizations and long-term hospital stay (Table 2).

**Table 2:** Follow-up comparisons between groups.

	Total	Chagas	Non-Chagas	ρ
Death from any cause (%)	54.1	56.4	53.7	0.712
Sudden death (%)	22.5	14.5	24.0	0.122
Worsening heart failure death (%)	22.2	27.3	21.3	0.325
Other cardiovascular death (%)	3.8	5.5	3.5	0.484
Stroke (%)	0.9	3.6	0.3	0.017
Noncardiovascular death (%)	0.9	3.6	0.3	0.017
Unknown (%)	4.7	5.5	4.5	0.766
Hospitalization				
Number, median (IQR)	0.0 (0.0–2.0)	1.0 (0.0–3.0)	0.0 (0.0–1.0)	0.005
Total days, median (IQR)	1.0 (0.0–22.0)	15.0 (0.0–48.0)	0.0 (0.0–20.0)	0.005
Need for emergency treatment				
Number, median (IQR)	2.0 (0.0–4.0)	2.0 (1.0–4.0)	2.0 (0.0–4.0)	0.655

Abbreviations: IQR, interquartile range.

### All-Cause, Heart Failure and Sudden Death Mortality Analysis

**Figure 2:**
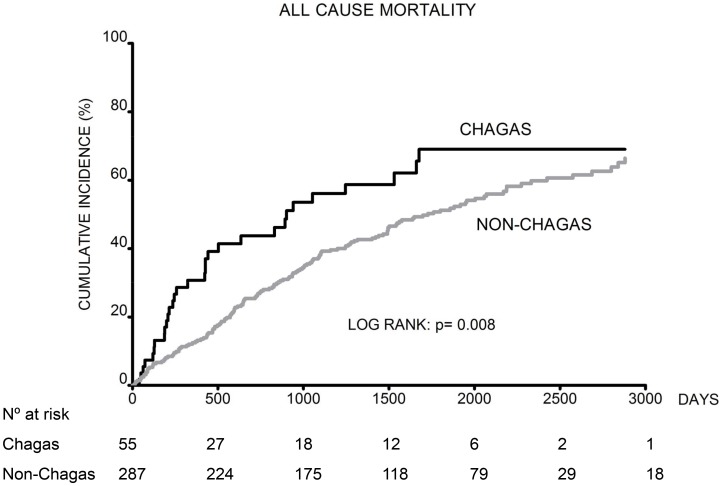
Cumulative incidence of all-cause mortality rate in Chagas and non-Chagas patients.

**Figure 3:**
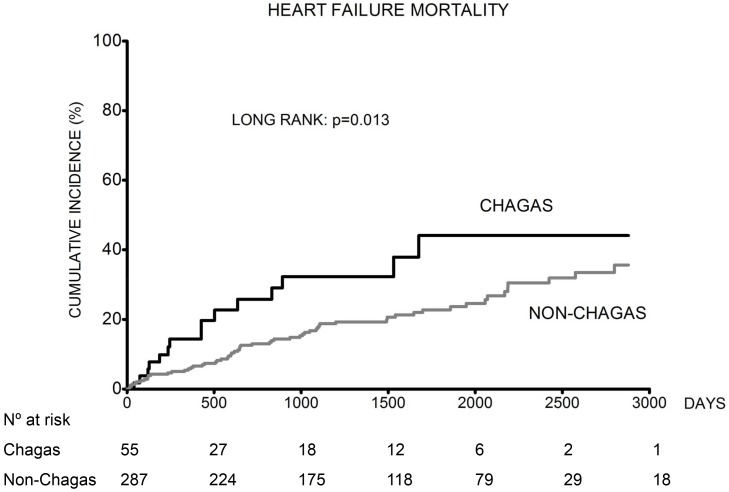
Cumulative incidence of heart failure mortality rate in Chagas and non-Chagas patients.

**Figure 4:**
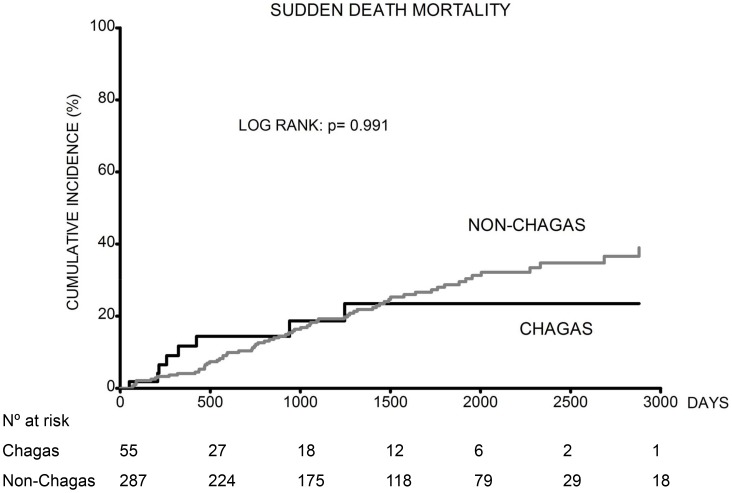
Cumulative incidence of sudden death mortality rate in Chagas and non-Chagas patients.

Altogether 185 (54.1%) patients died. Death occurred in 31 (56.4%) Chagas patients and 154 (53.7%) non-Chagas patients. Kaplan-Meier cumulative event curves for all-cause, heart failure and sudden death mortality are shown in Figures 2, 3 and 4.

The cumulative incidence of all-cause mortality was higher in patients with Chagas heart disease compared to non-Chagas patients (figure 2). Additionally, the cumulative incidence of heart failure mortality was higher in Chagas patients compared to non-Chagas (figure 3). Nevertheless, when we analyzed the cumulative incidence of sudden death mortality, there was no difference between the two groups (figure 4).

The influence of etiology on heart failure and sudden death mortality was further evaluated by Cox proportional-hazards regression. As we have information about New York Heart Association functional class (NYHA FC) only in 296 patients, we did two different models of Cox proportional-hazards regression; Model 1 without information about NYHA FC and Model 2 including NYHA FC (I/II vs III/IV). Other variables included were age, gender, etiology (Chagas vs non-Chagas), use of beta-blocker, use of angiotensin-converting enzyme inhibitor or angiotensin II receptor blocker, use of amiodarone, use of spironolactone, body mass index, left ventricular ejection fraction, left ventricular end diastolic diameter (LVEDD), group allocated (disease management program versus control), hemoglobin, serum sodium, creatinine clearance, number of hospitalizations, days of hospitalization and number of admissions to emergency care.

Chagas etiology, LVEDD, creatinine clearance and use of amiodarone were independently associated with heart failure mortality on Model 1 and Model 2 (table 3). On the other hand, LVEDD and use of beta-blocker were independently associated with sudden death mortality on both Models 1 and 2 (table 4).

**Table 3:** Cox proportional hazard analysis of risk factors at baseline for heart failure mortality.

	Univariate Analysis		Multivariate Analysis			
Variable	Unadjusted HR (95% Cl)	ρ	Model 1^h^, Adjusted HR (95% CI)	ρ	Model 2^h^, Adjusted HR (95% CI)	ρ
Age (years)		NS				
Male	1.655 (0.964–2.840)	0.065		NS		NS
BMI (kg/m^2^)		NS				
NYHA class^a^	1.593 (0.964–2.633)	0.069	NA			NS
LVEDD (mm)	1.038 (1.013–1.063)	0.003	1.071 (1.042–1.101)	<0.001	1.071 (1.039–1.104)	<0.001
LVEF (%)	0.027 (0.001–0.486)	0.015		NS		NS
Hemoglobin (g/dl)		NS				
CrCl (ml/min/1,73 m^2^)	0.991 (0.982–1.000)	0.038	0.986 (0.976–0.997)	0.009	0.984 (0.973–0.995)	0.006
Serum Sodium (mEq/l)	0.919 (0.857–0.985)	0.019		NS		NS
Etiology^b^	2.034 (1.150–3.599)	0.015	2.382 (1.204–4.711)	0.013	2.764 (1.343–5.688)	0.006
Beta-Blocker^c^		NS				
ACEI/ARB^d^		NS				
Spironolactone^e^		NS				
Amiodarone^f^	2.420 (1.304–4.492)	0.005	2.624 (1.326–5.192)	0.006	3.053 (1.469–6.343)	0.003
Group^g^		NS				
Number of hosp		NS				
Days of hosp	1.003 (1.001–1.005)	0.012		NS		NS
Number for EC		NS				

Abbreviations: ACEI, angiotensin-converting enzyme inhibitor; ARB, angiotensin II receptor blocker; BMI, body mass index; CrCl, creatinine clearance; EC, emergency care; Hosp, hospitalization; NYHA, New York Heart Association functional class; LVEF, left ventricular ejection fraction; LVEDD, left ventricular end diastolic diameter; NA: not applicable; NS: not significant;

a NYHA class III/VI vs NYHA class I/II;

b Chagas vs non Chagas;

c beta-blocker vs no beta-blocker;

d ACEI/ARB vs no ACEI/ARB;

e spironolactone vs no spironolactone;

f amiodarone vs no amiodarone;

g disease management program vs control;

h Model 1 not incorporated NYHA functional class and Model 2 included NYHA functional class (information available in 296 patients).

**Table 4:** Cox proportional hazard analysis of risk factors at baseline for sudden death mortality.

	Univariate Analysis		Multivariate Analysis			
Variable	Unadjusted HR (95% CI)	ρ	Model 1^h^, Adjusted HR (95% CI)	ρ	Model 2^h^, Adjusted HR (95% CI)	ρ
Age (years)		NS				
Male		NS				
BMI (kg/m^2^)		NS				
NYHA class^a^		NS	NA			
LVEDD (mm)	1.038 (1.012–1.064)	0.003	1.038 (1.010–1.065)	0.005	1.042 (1.013–1.072)	0.005
LVEF (%)	0.068 (0.004–1.259)	0.071		NS		NS
Hemoglobin (g/dl)		NS				
CrCl (ml/min/1,73 m^2^)		NS				
Serum Sodium (mEq/l)		NS				
Etiology^b^		NS				
Beta-Blocker^c^	0.617 (0.384–0.991)	0.046	0.586 (0.361–0.950)	0.030	0.521 (0.338–0.939)	0.014
ACEI/ARB^d^		NS				
Spironolactone^e^	1.696 (1.053–2.731)	0.030		NS		NS
Amiodarone^f^		NS				
Group^g^	1.710 (1.008–2.899)	0.047		NS		NS
Number of hosp		NS				
Days of hosp		NS				
Number for EC		NS				

Abbreviations: ACEI, angiotensin-converting enzyme inhibitor; ARB, angiotensin II receptor blocker; BMI, body mass index; CrCl, creatinine clearance; EC, emergency care; Hosp, hospitalization; NYHA, New York Heart Association functional class; LVEF, left ventricular ejection fraction; LVEDD, left ventricular end diastolic diameter; NA: not applicable; NS: not significant;

a NYHA class III/VI vs NYHA class I/II;

b Chagas vs non Chagas;

c beta-blocker vs no beta-blocker;

d ACEI/ARB vs no ACEI/ARB;

e spironolactone vs no spironolactone;

f amiodarone vs no amiodarone;

g disease management program vs control;

h Model 1 not incorporated NYHA functional class and Model 2 included NYHA functional class (information available in 296 patients).

Concerning the toxic effects of amiodarone, it is important to highlight that none patient died from lung toxicity.

### Severity of Heart Failure and Mode of Death

The relationship between mode of death and NYHA functional class at randomization was analyzed for each etiology. As mentioned before, information about NYHA functional class was available in 296 (87%) patients.

**Figure 5:**
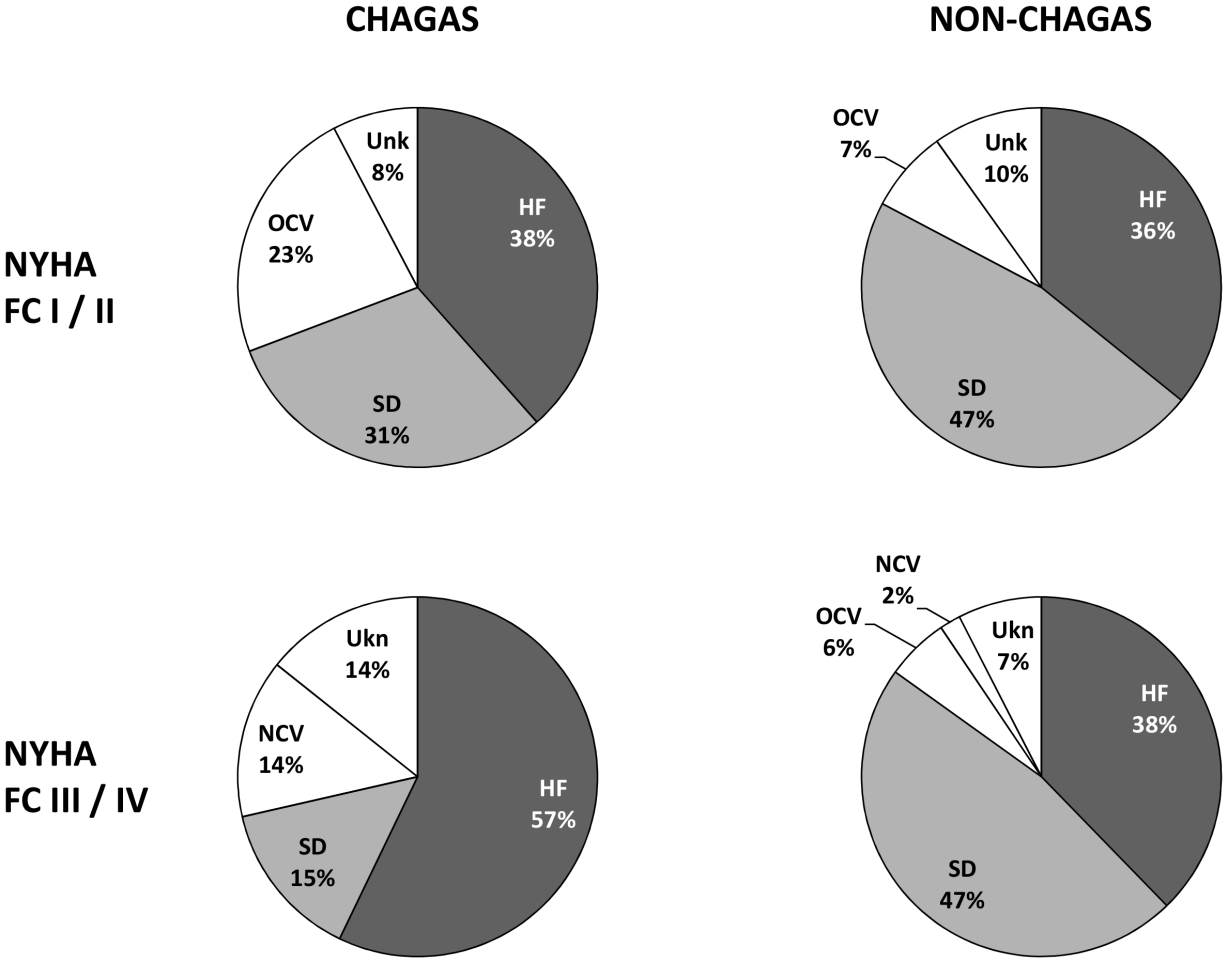
Severity of heart failure and mode of death in Chagas and non-Chagas patients. NYHA FC: New York Heart Association functional class; HF: heart failure; SD: sudden death; OCV: other cardiovascular death; NCV: non-cardiovascular death; Ukn: unknown.

In patients with Chagas heart disease, the proportion of patients who died of worsening heart failure increased substantially as increased functional class (38% *vs* 57%). On the other hand, the proportion of patients who died of sudden death decreased with the worsening of functional class (31% *vs* 15%). Furthermore, 23% of deaths in NYHA class I/II were secondary to other cardiovascular causes (Figure 5).

In non-Chagas group, sudden death was the main cause of death throughout the spectrum of NYHA functional class; 47% in both class I/II and class III/IV (figure 5).

## Discussion

To the best of our knowledge, this prospective study is the first one to investigate the causes of death in outpatients with Chagas heart disease associated with severe systolic dysfunction in comparison with non-Chagas patients. A recent study investigated risk estimation for cardiac events in Chagas disease but not analyzed the mode of death [10]. The present study found that patients with heart failure caused by Chagas heart disease had a worse prognosis when compared to others as well that the predominant mode of death in these chagasic patients were progressive heart failure throughout the spectrum of NYHA functional class presentation. In addition, the incidence of death related to cerebrovascular accident and non-cardiovascular deaths was higher in Chagas disease. In the same scenario, the main mode of death in non-Chagas group was sudden death. Moreover, in multivariate analysis Chagas heart disease, left ventricular end diastolic diameter, amiodarone use, serum sodium and creatinine clearance were independent risk factors for death from progressive heart failure.

Our finding is divergent from previous anecdotal reports concerning mode of death in chagasic heart failure patients that indicates sudden death as the main cause of death [4]. Prior studies had included few patients with severe heart failure or patients with higher values of left ventricular ejection fraction. Furthermore data were limited to abstracts or the treatments were outdated [4], [11]. Otherwise, other publications in Chagas disease had been selected patients with complex ventricular arrhythmias or patients under secondary prevention for sudden death [12], [13]. In the largest study in Chagas heart disease, 62.3% of the patient died of sudden death [14]. However the patients received outdated treatment characterized by low rate of beta-blockers (0.2%–2.4%) and of angiotensin-converting enzyme inhibitor (1.9%–21.9%), and unexplained high rates of amiodarone (35.1%–71.9%), that is not in accordance with guidelines for treatment of chagasic heart failure [15], [16]. As additional limitations, it was a retrospective study, the left ventricular function was not reported, only 10.4% of the patients were in NYHA functional class III/IV and the mode of death analysis was partially based on precarious Brazilian death certificate. Our results concerning progressive heart failure death are in concordance with other previous publications that included chagasic patients also under outdated treatment [17], [18]. Our finding of high incidence of stroke causing death in Chagas disease in comparison with other etiologies confirms previous studies [18], [19] despite the unexpected recently reported lack of evidence of pro-thrombotic status among patients with Chagas disease [20]. However, cardiac embolus originating from apical aneurysm and left ventricular thrombosis are a recurrent cause of Chagas stroke [21]. The explanation for high incidence of non-cardiovascular death on Chagas heart disease is unknown. However, in general chagasic patients have lower social and nutritional status and higher incidence of comorbidities, which could predispose them to non-cardiovascular death [22].

Some distinguishing clinical and anatomopathological aspects should be noted in Chagas disease that might influence the mode of death. Biventricular impairment with persistent myocarditis, inflammatory infiltrate edema, contraction-band necrosis and myocytolysis, focal and diffuse areas of myocellular hypertrophy, and fibrosis are found in histology of cardiac tissue [23]–[26]. Also, chronic low-grade parasite persistence drives tissue damage and the autoimmune component of Chagas cardiomyopathy [27]. Even though this autoimmunity may play a role in development of Chagas heart disease, this was never demonstrated in clinical setting. The role of anti-muscarinic antibodies in the determination of mode of death should be investigated [28]. Concerning the effects of chagasic myocarditis in the mode of death, our findings are in agreement with a recent study, which included 181 patients suspected of viral myocarditis followed for 58 months and did not show prevalence of sudden death as mode of death [29].

One would expect the influence of fibrosis in Chagas heart disease determining sudden death as the main mode of death as in ischemic cardiomyopathy. Although pathological findings in Chagas disease reported a pattern of diffuse interstitial fibrosis [30], magnetic resonance imaging study reported a pattern of fibrosis that can vary a lot from diffuse, heterogeneous, small focal to big transmural scars indistinguishable from myocardial infarction [31]. Ischemic cardiomyopathy displays regional fibrosis and scars which seem to have a mechanistic link with malignant ventricular arrhythmias [32]. The remarkable chagasic biventricular impairment, especially right-sided failure has been appointed as an independent predictor of survival in Chagas heart disease [18]. In fact, the complex Chagas heart disease is characterized by multiple clinical and anatomopathological factors that determine the mode of death, the clinical manifestation and the worst prognosis observed in our study as well as in other publications [33], [34].

The medical treatment might have influenced our results. The 38% rate of beta-blockers prescription to our chagasic patients cannot be considered substantial but it was in accordance of guidelines at the period of inclusion of REMADHE , when beta-blockers use was controversial in chagasic patients [35]. Only recently the use of beta-blockers was included in guidelines for the treatment of chagasic patients [15]. However, this 38% rate is considerably higher in comparison with previous studies where the rate of beta-blockers were under 15% [13], [14]. In addition, it could partially explain our results given that beta-blockers reduce sudden death in heart failure [36].

Our group has previously published that beta-blockers attenuate the worst prognosis of Chagas heart disease, approaching similar survival to other etiologies [33]. In this study, use of beta-blockers was not independently associated with the risk of death from worsening heart failure but it was independently associated with sudden death mortality. Based on our results, it is reasonable to suppose that beta-blockers had a more prominent effect in preventing sudden death than worsening heart failure death in patients with Chagas cardiomyopathy.

Specifically regarding the influence of amiodarone, which has been widely prescribed in Chagas heart disease to prevent sudden death and has reached up to 70% rate of use [13], [37], [38], our cohort had a lower rate of its use (9.1% in total cohort and 12.7% in Chagas group). Despite this, the percentage of sudden death was strikingly lower in this etiology. According to I Latin American Guidelines for the diagnosis and treatment of Chagas' heart disease, amiodarone is indicated only to (Class I) sustained ventricular tachycardia (symptomatic or not) or symptomatic non-sustained ventricular tachycardia and (Class IIb) for asymptomatic ventricular premature beats or non-sustained ventricular tachycardia [15].

On the other hand, amiodarone was an independent predictor of worsening heart failure death in our cohort, which agrees with previous study in non-chagasic population [39]. This finding could be related to a more strict indication of amiodarone in severe cases. However, this is an issue to be clarified in future studies because amiodarone prescription in our heart failure clinic is indicated according Brazilian Heart Failure Guidelines [16], in clinical situations not necessarily related to worst prognosis in heart failure.

In the face of our findings, an awkward question arises related to the higher incidence of sudden death in populations of chagasic patients despite the high rate of unexplained prescription of amiodarone [13], [40]. One issue to be clarified is the potential influence on mortality of amiodarone side effects such as pro-arrhythmic effects, bradycardia and AV block in a disease known to have high incidence of AV block, sick sinus syndrome, besides the increase deaths from circulatory failure and non-cardiovascular death [39], [41].

In non-Chagas group, our proportion of sudden death and progressive heart failure death is consistent with literature data. It is worth noting that our prescription of beta-blockers, angiotensin-converting enzyme inhibitor/angiotensin II receptor blocker and spironolactone reflects the state-of-the-art treatment of heart failure. Our results corroborate the EMPHASIS trial, which studied mild non-chagasic heart failure and demonstrated sudden death as main mode of death among cardiovascular death [42]. Also, the CIBIS trial reported sudden death as the predominant mode of death in the bisoprolol arm in non chagasic severe heart failure (NYHA class III/IV) [43].

### Limitations

This study contains some limitations that should be acknowledged. First, this study was a single center trial that included patients initially enrolled in another intervention trail. In addition, this was a retrospective analysis though the data were obtained prospectively in REMADHE trial.

Concerning our cohort, Chagas group is considerably smaller than non-Chagas group and Kaplan-Meier curves result may have limited value after 1500 days since the number of Chagas patients at risk is very low. However, it is important to note that the difference in proportion between groups reflects the reality of our population.

In few patients, the mode of death of patients was not clarified. However, it was less than 10%. Unfortunately, the gold standard autopsy studies were not available widespread to confirm certainly the cause of death. Therefore, in most randomized trials as in the REMADHE trial the autopsy is not routinely performed.

### Conclusions

Clinicians should keep in mind that in severe Chagas heart disease progressive heart failure plays an important role as the most important mode of death in a scenario of worse prognosis in comparison with other etiologies. The data from this analysis challenges the current understanding of Chagas heart disease and may have implications in the selection of treatment choices considering the mode of death. In addition, our results may influence the development of new strategies for prevention of chagasic heart failure. The amiodarone role in chagasic heart disease should be reevaluated. Moreover, prevention of thromboembolism must be emphatically pursued. Finally, priority should be given to enhance the research related to prevent and treat the progression of heart failure in Chagas disease.
